# Robust Facial Expression Recognition via Compressive Sensing

**DOI:** 10.3390/s120303747

**Published:** 2012-03-21

**Authors:** Shiqing Zhang, Xiaoming Zhao, Bicheng Lei

**Affiliations:** 1 School of Physics and Electronic Engineering, Taizhou University, Taizhou 318000, China; E-Mails: tzczsq@163.com (S.Z.); leibicheng@163.com (B.L.); 2 Department of Computer Science, Taizhou University, Taizhou 318000, China

**Keywords:** compressive sensing, sparse representation, facial expression recognition, Gabor wavelets representation, local binary patterns, corruption and occlusion

## Abstract

Recently, compressive sensing (CS) has attracted increasing attention in the areas of signal processing, computer vision and pattern recognition. In this paper, a new method based on the CS theory is presented for robust facial expression recognition. The CS theory is used to construct a sparse representation classifier (SRC). The effectiveness and robustness of the SRC method is investigated on clean and occluded facial expression images. Three typical facial features, *i.e*., the raw pixels, Gabor wavelets representation and local binary patterns (LBP), are extracted to evaluate the performance of the SRC method. Compared with the nearest neighbor (NN), linear support vector machines (SVM) and the nearest subspace (NS), experimental results on the popular Cohn-Kanade facial expression database demonstrate that the SRC method obtains better performance and stronger robustness to corruption and occlusion on robust facial expression recognition tasks.

## 1. Introduction

The traditional human computer interaction (HCI) system, in which a single user faces a computer and interacts with it via a mouse or a keyboard, were developed to emphasize the transmission of explicit messages while ignoring implicit information about the user, such as the user’s changes in the affective states. Such interactions are thus frequently perceived as incompetent, cold, and socially inept. This fact has inspired an active research field of “affective computing” [1], which aims at enabling computers to recognize, express, model, communicate and respond to a user’s emotion information. One of the most important applications of affective computing is to make HCI become more human-like, more effective, and more efficient. Specifically, such computers with the ability of affective computing could detect and track a user's affective states and initiate communications based on this information, rather than simply responding to a user’s commands.

Affective arousal modulates all nonverbal communication cues such as facial expression, vocal intonations, body gesture and movement. Facial expression is the most natural and efficient means for humans to communicate their emotions and intentions, as communication is primarily carried out face to face. During the past decade, facial expression recognition has attracted a significant interest in the scientific community, as it plays a vital role in the field of HCI.

Generally, a basic automatic facial expression recognition system consists of three steps [2]: face acquisition, facial feature extraction and representation, and facial expression recognition. Face acquisition is a preprocessing stage to automatically find the face regions in input images or sequences. Many face detection methods have been proposed to detect faces in an arbitrary scene. Viola and Jones [3] presented a robust real-time face detector based on a set of rectangle of features. Heisele *et al*. [4] developed a component-based framework to detect frontal and near-frontal views of faces in still gray images. El-Bakry [5] proposed a principal component analysis (PCA) based real-time face detection method by performing cross-correlation in the frequency domain between the input images and eigenvectors. The detected face regions are usually aligned based on the eye positions that can be detected in the face regions.

Facial feature extraction and representation aims to extract facial features to represent the facial changes caused by facial expressions. Two types of features, i.e., geometric features and appearance features, are usually used for facial representation [2]. Geometric features present the shape and locations of facial components such as mouth, eyes, brows, and nose. The facial components or facial feature points are extracted to form a feature vector that represents the face geometry. Fiducial facial feature points have been widely adopted as geometric features for facial representation. For instance, the geometric positions of 34 fiducial points on a face are usually used to represent facial images [6,7]. In contrast to geometric features, appearance features encode changes in skin texture such as wrinkles, bulges and furrows. The representative appearance features contains the raw pixels of facial images, Gabor wavelets representation [8,9], Eigenfaces [10], and Fisherfaces [11], *etc*. In recent years, a new face descriptor called local binary patterns (LBP) [12], have been widely used as appearance features for facial representation [13–16] due to its tolerance against illumination changes and computational simplicity.

Facial expression recognition is to use the extracted facial features to recognize different expressions. Depending on whether the temporal information is considered, facial expression recognition approaches can be categorized as frame-based or sequence-based. The frame-based method does not take the temporal information of input images into account, and use the extracted features from a single image to recognize the expression of that image. In contrast, the sequence-based method attempts to capture the temporal pattern in a sequence to recognize the expression for one or more images. So far, various classifiers, including artificial neural network (ANN) [17], the nearest neighbor (NN) or K-nearest neighbor (KNN) [18,19], support vector machines (SVM) [20], and so on, have been applied for frame-based expression recognition. For sequence-based expression recognition, the widely used techniques are hidden Markov models (HMM) [21], dynamic Bayesian networks [22], SVM [23].

Among the above mentioned three steps, facial expression recognition is the most critical aspect for any successful facial expression recognition system. The performance of a facial expression recognition system is mainly decided by a classifier. Therefore, designing a good classifier is a crucial step on facial expression recognition tasks.

The recently-emerged compressive sensing (CS) (also called compressive sampling) theory [24–26], which originally aims to address signal sensing and coding problems, has shown tremendous potential for other problems like pattern recognition [27,28]. Recently, Nagesh and Li [29] have successfully employed the CS theory to develop a promising technique for expression-invariant face recognition. Nevertheless, they did not exploit the performance of the CS theory on the robust classification of occluded facial expression images. Note that, in real-world sceneries, facial images are usually corrupted by noise or outliers, that is, some pixels that do not belong to the facial images are depicted. Therefore, a study on robust facial expression recognition is more practical and meaningful. In addition, most existing facial expression recognition systems [13–23] focus on expression classification on clean facial images without any corruption. Motivated by little studies on the robust classification of facial expression with the CS theory, in this paper a new method of robust facial expression recognition based on the CS theory is presented.

The remainder of this paper is organized as follows: Section 2 gives the background and related work. In Section 3, facial feature extraction, including Gabor wavelets representation and local binary pattern (LBP), is reviewed briefly. The experiment verification is presented in detail in Section 4. Section 5 gives the conclusions.

## 2. Background and Related Work

In this section, we briefly review the CS theory, and then present the details of the recently-emerged sparse representation classifier (SRC) based on the CS theory.

### 2.1. Compressive Sensing (CS)

Given a system of under-determined equation:

(1) $$y_{m\times 1}=A_{m\times n}x_{n\times 1},\;m<n$$

Its known that the above Equation (1) has no unique solution, since the number of variables is larger than the number of equations. In signal processing terms, the length of the signal (*n*) is larger than the number of samples (*m*). However, according to the CS theory, if the signal is sparse, it is necessarily unique, and can be reconstructed by practical algorithms.

Suppose that the signal is *k*-sparse if it is a linear combination of only *k* basis vectors. That is, there are only *k* non-zero values in *x*, and the remainder are all zeroes. In this case, it is possible to find the solution to Equation (1) by a brute force enumeration of all the possible *k*-sparse vectors of length *n*. Mathematically speaking, this problem can be expressed as:

(2) $$\text{min}{\left\|x\right\|}_{0},\;\text{subject to}\;y=Ax$$

where ‖ ‖_0_ is the *l*_0_-norm and denotes the number of non-zero elements in the vector. Equation (2) is known to be an NP(non-deterministic polynomial) hard problem, and is thus not a practical solution to Equation (1). The CS literatures [24–26] indicates that under a certain condition on the projection matrix **A**, *i.e*., restricted isometry property (RIP), the sparsest solution to Equation (1) can be obtained by replacing the *l*_0_-norm in Equation (2) by its closest convex surrogate, the *l*_1_-norm (‖ ‖_1_). Therefore, the solution to Equation (2) is equivalent to the following *l*_1_-norm minimization problem:

(3) $$\text{min}{\left\|x\right\|}_{1},\;\text{subject to}\;y=Ax$$

where the *l*_1_-norm, ‖ ‖_1_, denotes the minimization of the sum of absolute values of elements in the vector, and serves as an approximation of the *l*_0_-norm.

In practice, the equality *y =* **A***x* is often relaxed to take into account the existence of measurement error in the sensing process due to a small amount of noise. Suppose the measurements are inaccurate and consider the noisy model:

(4) y=Ax+e

where *e* is a stochastic or deterministic error term. Particularly, if the error term *e* is assumed to be white noise such that ‖*e*‖_2_ < *ε*, where *ε* is a small constant. A noise robust version of Equation (3) is defined as follows:

(5) $$\text{min}{\left\|x\right\|}_{1},\;\text{subject to}\;{\left\|y-Ax\right\|}_{2}<\varepsilon$$

To solve the *l*_1_-minimization of Equations (3) and (5), various efficient algorithms have been developed. Two typical algorithms based on the interior-point idea, are l1-magic [30] and l1-ls [31]. The l1-magic algorithm [30] recasts the *l*_1_-minimization problem as a second-order cone program and then applies the primal log-barrier approach. The l1-ls algorithm [31] is a specialized interior-point method for solving the large-scale 11-regularized least-squares programs that uses the preconditioned conjugate gradients algorithm to compute the search direction.

### 2.2. Sparse Representation Classifier (SRC)

Recently, a sparse representation classifier (SRC) has been developed based on the CS theory [27,28]. In the SRC algorithm, it is assumed that the whole set of training samples form a dictionary, and then the recognition problem is cast as one of discriminatively finding a sparse representation of the test image as a linear combination of training images by solving the optimization problem in Equation (3) or (5). Formally, for the training samples of a single class, this assumption can be expressed as:

(6) $$y_{k,\mathrm{test}}=\alpha_{k,1}\;y_{k,1}+\alpha_{k,2}\;y_{k,2}+\cdots +\alpha_{k,n_{k}}\;y_{k,n_{k}}+\varepsilon_{k}=\sum_{i=1}^{n_{k}}\alpha_{k,i}\;y_{k,i}+\varepsilon_{k}$$

where *y*_*k*,*test*_ is the test sample of the *k^th^* class, *y_k_*_,_*_i_* is the *i^th^* training sample of the *k^th^* class, *α_k_*_,_*_i_* is the weight corresponding weight and *ε_k_* is the approximation error.

For the training samples from all *c* object classes, the aforementioned Equation (6) can be expressed as:

(7) $$\begin{array}{ll} y_{k,\mathrm{test}} & =\alpha_{1,1}\;y_{1,1}+\cdots +\alpha_{k,1}\;y_{k,1}+\cdots +\alpha_{k,n_{k}}\;y_{k,n_{k}}+\cdots +\alpha_{c,n_{c}}\;y_{c,n_{c}}+\varepsilon \\ & =\sum_{i=1}^{n_{1}}\alpha_{1,i}\;y_{1,i}+\cdots +\sum_{i=k}^{n_{k}}\alpha_{k,i}\;y_{k,i}+\cdots +\sum_{i=1}^{n_{c}}\alpha_{c,i}\;y_{c,i}+\varepsilon \end{array}$$

In matrix-vector notation, Equation (7) can be rewritten as:

(8) $$y_{k,\mathrm{test}}=A\alpha +\varepsilon$$

where $\left\{ \begin{array}{l} A=[y_{1,1}\left|\cdots \right|y_{1,n_{1}}\left|\cdots \right|y_{k,1}\left|\cdots \right|y_{k,n_{k}}\left|\cdots \right|y_{c,1}\left|\cdots \right|y_{c,n_{c}}]\\ \alpha ={\left[\alpha_{1,1}\cdots \alpha_{1,n_{1}}\cdots \alpha_{k,1}\cdots \alpha_{k,n_{k}}\cdots \alpha_{c,1}\cdots \alpha_{c,n_{c}}\right]}^{\prime } \end{array}\right.$

The linearity assumption in the SRC algorithm coupled with Equation (8) implies that the weight vector **α** should be zero except those associated with the correct class of the test sample. To obtain the weight vector **α**, the following *l*_0_-norm minimization problem should be solved:

(9) $$\underset{\alpha }{\text{min}}{\left\|\alpha \right\|}_{0},\;s.t.\;{\left\|y_{k,\mathrm{test}}-A\alpha \right\|}_{2}\leq \varepsilon$$

It is known that Equation (9) is an NP-hard problem. The NP-hard *l*_0_-norm can be replaced by its closest convex surrogate, the *l*_1_-norm. Therefore, the solution of Equation (9) is equivalent to the following *l*_1_-norm minimization problem:

(10) $$\underset{\alpha }{\text{min}}{\left\|\alpha \right\|}_{1},\;s.t.\;{\left\|y_{k,\mathrm{test}}-A\alpha \right\|}_{2}\leq \varepsilon$$

This is a convex optimization problem and can be solved by quadratic programming. Once a sparse solution of **α** is obtained, the classification procedure of SRC is summarized as follows:

Step 1: Solve the *l*_1_-norm minimization problem in Equation (10).
Step 2: For each class *i*, compute the residuals between the reconstructed sample $y_{\text{recons}}\;(i)=\sum_{j=1}^{n_{i}}\alpha_{i,j}\;y_{i,j}$ and the given test sample by *r*(*y_test_, i*) = ‖*y_k,test_* − *y_recons_*(*i*)‖_2_.
Step 3: The class of the given test sample is determined by identify (*y_test_*) = arg min*_i_ r*(*y_test_, i*).

## 3. Facial Feature Extraction

In this section, two types of facial feature extraction: Gabor wavelets representation and local binary pattern (LBP), are briefly introduced.

### 3.1. Gabor Wavelets Representation

Gabor wavelets model quite well the receptive field properties of cells in the primary visual cortex [8,9]. The Gabor wavelets kernels exhibit strong characteristics of spatial locality and orientation selectivity, making them a suitable choice for image feature extraction when one’s goal is to derive local and discriminating features for facial expression classification. The Gabor wavelet kernels can be defined as:

(11) $$\varphi_{\mu ,\nu }\;(z)=\frac{{\left\|k_{\mu ,\nu }\right\|}^{2}}{\sigma^{2}}e^{-\frac{{\left\|k_{\mu ,\nu }\right\|}^{2}{\left\|z\right\|}^{2}}{{2\sigma }^{2}}}[e^{{\mathrm{ik}}_{\mu ,\nu }z}-e^{-\frac{\sigma^{2}}{2}}]$$

where *μ* and *ν* denote the orientation and scale of the Gabor kernel, *z* = (*x*, *y*), ‖·‖ denotes the norm operator, and the wave vector *k_μ,ν_* is defined as:

(12) $$k_{\mu ,\nu }=k_{\nu }e^{i\phi_{\mu }}$$

where *k*_ν_ = *k*_max_/ *f^ν^* and *ϕ_μ_* = *πμ*/8. *k*_max_ is the maximum frequency, and *f* is the spacing factor between kernels in the frequency domain.

**Figure 1.**
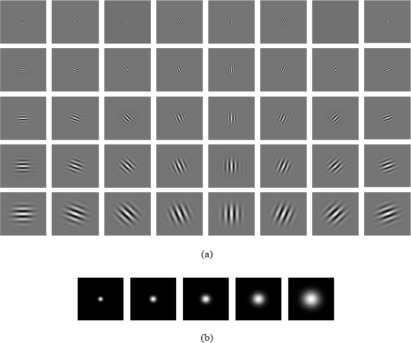
(**a**) The real part of the Gabor wavelet kernels at five scales and eight orientations; (**b**) The magnitude of the Gabor wavelet kernels at five scales.

As done in [20,32], we used 40 Gabor wavelet kernels at five scales, *ν* = {0,1, ⋯, 4}, and eight orientations, *μ* = {0,1, ⋯, 7}, with *σ* = 2*π*,*k*_max_ = *π*/2, and $f=\sqrt{2}$. Figure 1 shows the real part of the Gabor wavelet kernels at five scales and eight orientations, and their magnitudes. The Gabor wavelets representation is essentially the concatenated pixels of the 40 modulus-of-convolution images obtained by convolving the input image with these 40 Gabor kernels. In practice, the magnitude of Gabor wavelets representation is used for facial expression recognition. As suggested in [33], before concatenation each output image is down-sampled by a factor of 16 and normalized to zero mean and unit variance.

### 3.2. Local Binary Patterns

The local binary pattern (LBP) operator [12] is a gray-scale invariant texture primitive statistic, which has shown excellent performance in the classification of various kinds of textures. For each pixel in an image, a binary code is produced by thresholding its neighborhood with the value of the center pixel. The LBP code of the center pixel in the neighborhood is obtained by converting the binary code into a decimal one. Based on the LBP operator, each pixel of an image is labeled with an LBP code. The 256-bin histogram of the labels contains the density of each label and can be used as a texture descriptor of the considered region.

**Figure 2.**
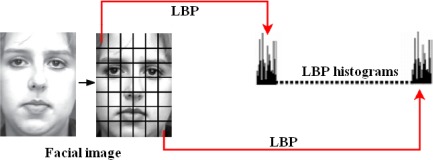
The process of LBP features extraction

The process of LBP features extraction is summarized as follows: firstly, a facial image is divided into several non-overlapping blocks. Secondly, LBP histograms are computed for each block. Finally, the block LBP histograms are concatenated into a single vector. As a result, the facial image is represented by the LBP code. Figure 2 presents the process of LBP features extraction.

## 4. Experiment Verification

To verify the effectiveness and robustness of SRC on facial expression recognition tasks, the popular Cohn-Kanade database [34], are used for experiments. Three typical facial features, including the raw pixels, Gabor wavelets representation and local binary patterns (LBP), are extracted to testify the performance of SRC on facial expression recognition tasks. To reduce the feature length of Gabor wavelets representation, principal component analysis (PCA) [35] is used for dimensionality reduction. The reduced feature dimension is confined to the range (0, 100) with an interval of 10. The performance of SRC is compared with the nearest neighbor (NN), linear SVM as well as the recently developed non-parametric nearest subspace (NS) method [36]. Note that, for the SRC method, it’s necessary to normalize the training and testing data with unit *l*_2_-norm. The experiment platform is Intel CPU 2.10 GHz, 1 G RAM memory, MATLAB 7.0.1 (R14).

A 10-fold cross validation scheme is employed in 7-class facial expression recognition experiments, and the average recognition results are reported. In detail, each classification model is trained on nine tenths of the total data and tested on the remaining tenth. This process is repeated ten times, each with a different partitioning seed, in order to account for variance between the partitions. We provide facial expression recognition results and analysis in two aspects. On one hand, facial expression recognition experiments are directly performed on original clean images without any occlusion. On the other hand, facial expression recognition experiments are conducted when the random pixel corruption and the random block occlusion occur in the test images resized with 32 × 32 pixels.

### 4.1. Database and Pre-Processing

**Figure 3.**
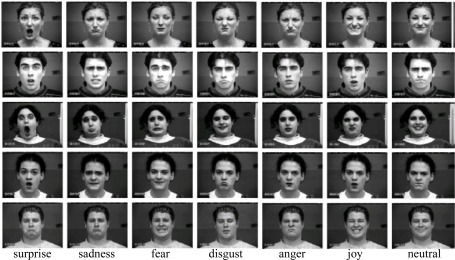
Examples of facial expression images from the Cohn-Kanade database.

The Cohn-Kanade database [34] consists of 100 university students aged from 18 to 30 years, of which 65% were female, 15% were African-American and 3% were Asian or Latino. Subjects were instructed to perform a series of 23 facial displays, six of which were based on description of prototypic emotions. Image sequences from neutral to target display were digitized into 640 × 490 pixels with 8-bit precision for grayscale values. Figure 3 shows some sample images from the Cohn-Kanade database. In this work, 320 image sequences were selected from the Cohn-Kanade database. The selected sequences, each of which could be labeled as one of the six basic emotions, come from 96 subjects, with 1 to 6 emotions per subject. For each sequence, the neutral face and one peak frames were used for prototypic expression recognition. Finally, 470 images (32 anger, 100 joy, 55 sadness, 75 surprise, 47 fear, 45 disgust and 116 neutral) were obtained for experiments.

For the raw pixels extraction, the size of original facial images is directly down-sampled to 32 × 32 pixels. The only reason for resizing the image with 32 × 32 pixels is that all the experiments can be performed within the memory size of MATLAB on a typical PC.

For Gabor wavelets representation and LBP features extraction, our pre-processing is similar to that used in [13,15]. We normalized the eye distance of facial images to a fixed distance of 55 pixels once the centers of two eyes were located. Generally, it is observed that the width of a face is roughly two times of the distance, and the height is roughly three times. Therefore, based on the normalized value of the eye distance, a resized image of 110 × 150 pixels was cropped from an original image.

The cropped facial images of 110 × 150 pixels contain facial main components such as mouth, eyes, brows and noses. The Gabor wavelets representation is obtained by convolving the whole region of the cropped facial image with the Gabor kernels. Likewise, the LBP features are obtained by applying the LBP operator to the whole region of the cropped facial images. Similar to the settings in [13–15,38], we selected the 59-bin operator, ${\mathrm{LBP}}_{P,R}^{u2}$, where the notation (*P*, *R*) denotes a neighborhood of *P* equally spaced sampling points on a circle of radius of *R* that form a circularly symmetric neighbor set, and the superscript u2 in ${\mathrm{LBP}}_{P,R}^{u2}$ indicates using only uniform patterns and labeling all remaining patterns with a single label. And then we divided the 110 × 150 pixels facial images into 18 × 21 pixels regions, giving a good trade-off between recognition performance and feature vector length. Thus facial images were divided into 42 (6 × 7) regions, and represented by the LBP histograms with the length of 2,478 (59 × 42).

### 4.2. Experimental Results without Occlusion

**Figure 4.**
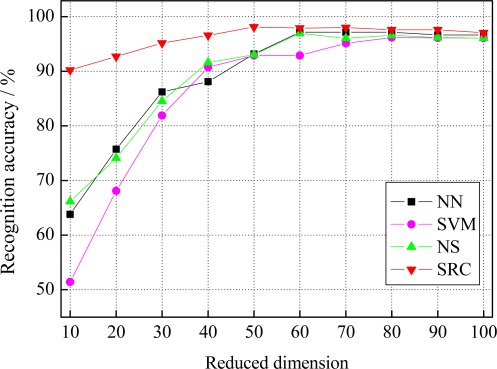
Recognition results of different methods with reduced dimension of Gabor wavelets representation.

When using the raw pixels (*i.e*., the resized images of 32 × 32 pixels) and LBP features for experiments, the corresponding recognition results and standard deviations (std) of different methods, including NN, SVM, NS, as well as SRC, are given in Table 1. The recognition results of different methods along with reduced dimension of Gabor wavelets representation are presented in Figure 4. Table 2 shows the best accuracy of different methods with the corresponding reduced dimension of Gabor wavelets representation. The results in Tables 1–2 and Figure 4 reveal that SRC achieves an accuracy of 94.76% with the raw pixels, 97.14% with LBP features, and 98.1% at best with 50 reduced dimension of Gabor wavelets representation, outperforming the other used methods. This confirms the validity and high performance of SRC for facial expression recognition.

Tables 3–5 displays the confusion matrix of recognition results of SRC with the raw pixels, LBP features, and Gabor wavelets representation, respectively. From the results in Tables 3–5, we can see that most of seven expressions are identified very well with an accuracy of 100%.

The obtained recognition accuracy of SRC (*i.e*., 97.14% with LBP features, and 98.1% with Gabor wavelets representation) on 7-class facial expression recognition tasks is highly competitive, compared to previously reported results on the Cohn-Kanade database. In [14], on 7-class facial expression recognition tasks they employed LBP-based SVM to give the best accuracy of 88.4%. In [13], with LBP features and SVM they reported a 7-class recognition accuracy of 91.4% at best. In [37], they obtained the highest accuracy of 93.4% with SVM on 7-class tasks, but they used an improved LBP features called local directional pattern (LDP).

### 4.3. Experimental Results with Occlusion

In this section, we used the resized image of 32 × 32 pixels from the Cohn-Kanade database to verify the robustness of SRC to two kinds of occlusions, *i.e*., the random pixel corruption and the random block occlusion.

**Figure 5.**
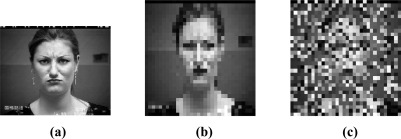
A corrupted image example (**a**) Original image of 640 × 490 pixels; (**b**) Resized image of 32 × 32 pixels; (**c**) 50% corrupted image.

**Figure 6.**
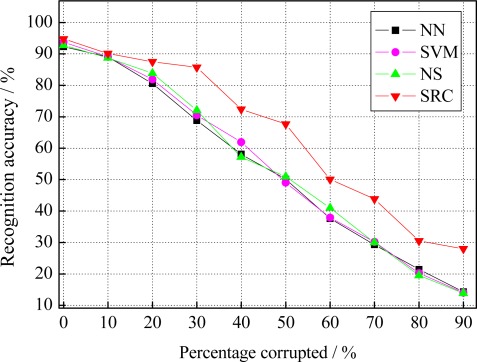
Recognition accuracy under different percentage corrupted.

At first, we consider the recognition of facial expressions with different percentage of image pixels corrupted at random. The percentage of the pixels are randomly chosen from each of test image and replaced by random values in the range [0, M_i_], where M_i_ is the maximum pixel value in the *i*th test image. The percentage of corrupted pixels varies from 0% to 90%. Figure 5 gives an example of a 50% corrupted face image on the resized image of 32 × 32 pixels. As shown in Figure 5, beyond 50% corruption, the corrupted images are scarcely identified as facial images. Figure 6 plots the recognition accuracy of all used methods, *i.e*., NN, SVM, NS and SRC, under different percentage corrupted from 0% to 90%. It can be observed that the performance of all used methods decreased as the percentage corrupted increased. Nevertheless, SRC still dramatically outperforms the other used methods at various levels of corruption. This indicates SRC is more robust to the random pixels corruption than the other used methods.

**Figure 7.**
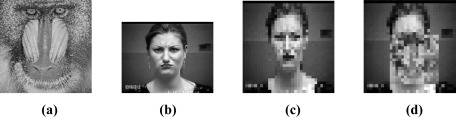
An occluded image example (**a**) Baboon; (**b**) Original image of 640 × 490 pixels; (**c**) Resized image of 32 × 32 pixels; (**d**) 30% occluded image.

**Figure 8.**
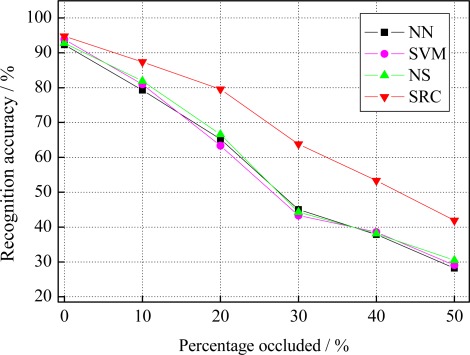
Recognition accuracy under different percentage occluded.

We next investigate the robustness of SRC to the random block occlusion. We simulate this situation under different percentage occluded, from 0% to 50%, by replacing a randomly located square block of each test image with an unrelated image of a baboon, as shown in Figure 7(a). Note that, the location of occlusion is randomly chosen for each image and is unknown to the algorithm. Figure 7 shows an example of a 30% occluded face image. To the human eye, beyond 30% occlusion, the entire facial regions have been almost completely occluded. In this case, it’s a difficult recognition task even for humans. Figure 8 gives the recognition performance of SRC and its three competitors, as a function of the percentage occluded from 0% to 50%. As illustrated in Figure 8, we can see that the recognition accuracy of SRC significantly exceeds that of other used methods at various levels of occlusion. This demonstrates SRC achieves a higher level of robustness to the random block occlusion in comparison with the other used methods.

## 5. Conclusions

In this paper, we present a new technique of robust facial expression recognition via sparse representation classifier (SRC) based on the CS theory. Experimental results on the popular Cohn-Kanade facial expression database demonstrate that SRC obtains promising performance on facial expression recognition without occlusion, and exhibits a strong robustness to the random pixel corruption and the random block occlusion occurred in facial expression images. It’s worth pointing out that in this work for simplicity we only focus on the static 2D facial expression recognition. In recent years, 3D facial expression recognition has been considered as a major solution to handle the unsolved issues of reliable 2D facial expression recognition, *i.e*., illumination and pose changes. Therefore, it’s also an interesting task to investigate the performance of the presented method for 3D facial expression recognition.

## Figures and Tables

**Table 1.** Recognition results (%) of different methods with the raw pixels and LBP features.

**Methods**	**NN**	**SVM**	**NS**	**SRC**
Raw pixels	92.29±1.9	93.80±2.1	92.74±1.9	94.76±1.7
LBP	96.22±4.6	95.24±4.2	95.71±5.8	97.14±3.9

**Table 2.** Best results (%) of different methods with reduced dimension of Gabor wavelets representation.

**Methods**	**NN**	**SVM**	**NS**	**SRC**
Dimension	60	80	60	50
Accuracy	97.14±3.7	96.17±4.0	96.94±4.3	98.10±3.8

**Table 3.** Confusion matrix of recognition results of SRC with the raw pixels.

	**Anger (%)**	**Joy (%)**	**Sadness (%)**	**Surprise (%)**	**Disgust (%)**	**Fear (%)**	**Neutral (%)**
Anger	**90**	10	0	0	0	0	0
Joy	0	**100**	0	0	0	0	0
Sadness	0	0	**90**	0	10	0	0
Surprise	0	0	0	**100**	0	0	0
Disgust	0	0	0	0	**100**	0	0
Fear	0	0	0	0	0	**100**	0
Neutral	0	0	6.67	0	3.33	6.67	**83.33**

**Table 4.** Confusion matrix of recognition results of SRC with LBP features.

	**Anger (%)**	**Joy (%)**	**Sadness (%)**	**Surprise (%)**	**Disgust (%)**	**Fear (%)**	**Neutral (%)**
Anger	**90**	0	0	0	0	0	10
Joy	0	**100**	0	0	0	0	0
Sadness	3.33	0	**90**	0	0	0	6.67
Surprise	0	0	0	**100**	0	0	0
Disgust	0	0	0	0	**100**	0	0
Fear	0	0	0	0	0	**100**	0
Neutral	0	0	0	0	0	0	**100**

**Table 5.** Confusion matrix of recognition results of SRC with 50 reduced Gabor wavelets representation.

	**Anger (%)**	**Joy (%)**	**Sadness (%)**	**Surprise (%)**	**Disgust (%)**	**Fear (%)**	**Neutral (%)**
Anger	**100**	0	0	0	0	0	0
Joy	0	**100**	0	0	0	0	0
Sadness	0	0	**100**	0	0	0	0
Surprise	0	0	0	**96.67**	0	0	3.33
Disgust	10	0	0	0	**90**	0	0
Fear	0	0	0	0	0	**100**	0
Neutral	0	0	0	0	0	0	**100**
